# Plant Extracts Obtained with Green Solvents as Natural Antioxidants in Fresh Meat Products

**DOI:** 10.3390/antiox10020181

**Published:** 2021-01-27

**Authors:** Mirian Pateiro, Julián Andrés Gómez-Salazar, Mariana Jaime-Patlán, María Elena Sosa-Morales, José M. Lorenzo

**Affiliations:** 1 Centro Tecnológico de la Carne de Galicia, Rúa Galicia Nº 4, Parque Tecnológico de Galicia, San Cibrao das Viñas, 32900 Ourense, Spain; mirianpateiro@ceteca.net; 2 Departamento de Alimentos, División de Ciencias de la Vida, Campus Irapuato-Salamanca, Universidad de Guanajuato, Irapuato, Guanajuato 36500, Mexico; julian.gomez@ugto.mx (J.A.G.-S.); m.jaimepatlan@ugto.mx (M.J.-P.); msosa@ugto.mx (M.E.S.-M.); 3 Área de Tecnología de los Alimentos, Facultad de Ciencias de Ourense, Universidad de Vigo, 32004 Ourense, Spain

**Keywords:** non-cooked meat products, natural preservatives, sustainable extraction, co-products, polyphenols

## Abstract

Plants are rich in bioactive compounds (BACs), mainly polyphenols, which are valuable choices to replace synthetic antioxidants in meat products. These natural antioxidants from plants, in the form of extracts and essential oils (EOs), have been obtained from different sources such as fruits (dragon fruit, guarana, pomegranate), vegetables, (cabbage, onion), herbs, and spices (epazote, ginger, rosemary, sage, thyme, turmeric, winter savory) by several extraction processes. However, in the context of current directives there is a notable incentive for “green” solvents to replace organic ones and conventional techniques, in order to avoid harm to the environment, operator, and consumer health. In addition, the recycling of co-products from the processing of these plant materials allow us to obtain valuable BACs from under-exploited materials, contributing to the revalorization of these wastes. The resulting extracts allow us to maintain the quality of meat products, exhibiting similar or better antioxidant properties compared to those shown by synthetic ones. Their incorporation in fresh meat products would maintain the oxidative stability, stabilizing colour parameters, decreasing the formation of metmyoglobin, lipid, and protein oxidation and the generation of lipid-derived volatile compounds, without affecting sensory attributes. In addition, these novel ingredients contribute to improve both technological and functional characteristics, thus diversifying the offer of so-called “wellness foods”. In this review, the application of plant extracts as natural antioxidants in several fresh meat products is presented, showing their efficacy as scavenging radicals and imparting additional health benefits.

## 1. Introduction

Meat and meat products are an excellent source of essential nutrients with high-quality proteins, carbohydrates, minerals, and pigments, and depending on the muscle type, contain variable quantities and proportions of storage (triacylglycerols) and structural lipids (phospholipids) [1,2,3,4,5]. Bacterial growth and lipid oxidation are mainly responsible for quality deterioration of meat products during storage, processing, and handling, reducing their shelf life and impairing their consumption [6,7,8].

Oxidation is one of the most important non-microbial degradation mechanisms of meat [9], which affects attributes such as taste, colour, texture, and nutritional value [10,11]. In meat, oxidation can be initiated endogenously via metallic ions, especially heminic iron, and via exogenous reactive oxygen species. Many factors, including animal species, breed, muscle type, diet, health, and post-slaughter processes can influence this reaction [12]. Lipid oxidation is quite a complex process whereby the unsaturated fatty acid fraction of membrane phospholipids is oxidized and hydroperoxides are formed, which are further susceptible to oxidation or decomposition to secondary oxidation products, such as short-chain aldehydes, ketones, and other oxidized compounds, such as malonaldehyde, that can be harmful to health and may adversely affect the overall quality and the acceptability of meat and meat products [6,11,13]. As a result, there is an increase in metmyoglobin (MetMb), thiobarbituric acid reactive substances (TBARS), and total bacterial counts in meat samples after storage, whereas pH, lightness, and redness values tend to decrease with increasing storage time [14].

Food degradation can be inhibited using additives, such as antioxidant compounds, which prevent oxidative changes in food by protecting it from free radicals [15]. Antioxidants can be added to meat and meat products during processing to delay these reactions [2], being the synthetic antioxidants commonly used in the meat industry since they are very effective against oxidation reactions [16]. However, controversy has arisen regarding their use due to recent studies that demonstrate the possible toxic effects of these additives [16,17]. In this regard, consumers are increasingly demanding new food options, especially those that contain natural and biologically active ingredients with the capacity to promote health and that are free of additives [18,19,20]. The demand for healthier products represents the major trend worldwide in industries, which have been seeking new ways to reduce the use of chemical additives, replacing them with natural alternatives. Therefore, the use of natural antioxidants in meat products seems a good option to reduce the consumption of synthetic additives [10,11,20,21]. In this regard, several plant extracts and essential oils (EOs) from diverse sources, such as aromatic plants, fruits, leaves, seeds, and spices, can be used as natural antioxidants in meat products, as they can retard or inhibit lipid and protein oxidation by preventing oxidative chain reactions and can extend the shelf life of these products [11,22,23,24,25].

Therefore, there is increasing use of plant extracts to replace chemical products in foods, especially in high-fat and ready-to-eat meat products [26]. The present review aims to present a comprehensive literature review on the use of natural antioxidants extracted by eco-innovative technologies in fresh meat products, evaluating the oxidative processes that occur in these foods. Their evaluation will allow us to know the real behaviour of these compounds, avoiding the interferences caused by cooking, which leads to an intense pro-oxidant environment in which both lipids and proteins can be affected.

## 2. Plants as a Natural Source of Antioxidants

Fruits, vegetables, spices, herbs, cereals, grains, and seeds are the major sources of plant-derived antioxidants. It is also important to note that the co-products generated during the processing of these foods can be also a source of antioxidants whose use would allow their revalorization, avoiding the important environmental problems and economic losses associated with these wastes [27,28].

**Figure 1:**
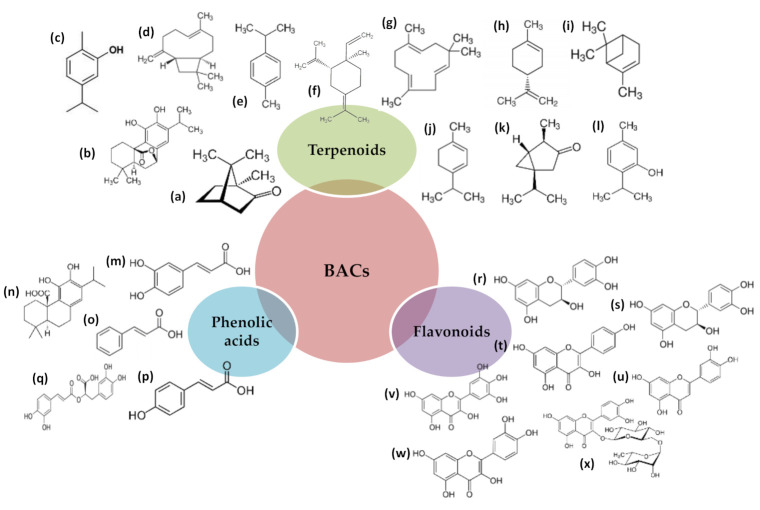
Main bioactive compounds (BACs) present in different plants extracts. (**a**). Camphor, (**b**). Carnosol, (**c**). Carvacrol, (**d**). β-Caryophyllene, (**e**). ρ-Cymene, (**f**). γ-Elemene, (**g**). α-Humelene, (**h**). Limonene, (**i**). α-Pinene, (**j**)**.** α-Terpinene, (**k**)**.** α-Thujone, (**l**). Thymol, (**m**). Caffeic acid, (**n**). Carnosic acid, (**o**). *trans*-Cinnamic acid, (**p**). ρ-Coumaric acid, (**q**). Rosmarinic acid, (**r**). Catechin, (**s**). Epicatechin, (**t**). Kaempferol, (**u**). Luteolin, (**v**). Myrcetin, (**w**). Quercetin, (**x**). Rutin.

The activity of the extracts and EOs obtained from these plant materials is mainly linked to the presence in their composition of compounds with strong antioxidant activity, mainly polyphenols and terpenoids [2,3,7,16,21]. Within each of them, anthocyanins, flavonols, and tannins or terpenes are usually the most abundant (Figure 1) [20]. Furthermore, it is important to note that the benefits ascribed to plant extracts cannot be attributed to a single class of compounds, but to the multiple contribution of different bioactive compounds (BACs). Among terpenes, compounds such as camphor, camphene, carnosol, carvacrol, thymol, α-pinene, ρ-cymene, 1,8-cineol, limonene, γ-terpinene, and terpinen-4-ol are the most common. Quercetin and kaempferol are the predominant flavonols, although others such as apigenin, isorhamnetin, luteolin, myricetin, and rutin also stand out [29]. Finally, phenolic acids such as caffeic, cinnamic, chlorogenic, ferulic, quinic, rosmarinic, and sinapic acids are among the most identified [30].

On the other hand, the type of compound and its mechanism of action are key factors to explain the antioxidant activity [31]. In this regard, the number and positions of the hydroxyl groups in association with methoxy and carboxylic acid groups are crucial to understand their activity. Moreover, this chemotype also depends on the part of the plant selected, the method of extraction, and the harvest season [32,33].

In addition to their known antioxidant activity and with respect to the objective of this review, these BACs have also shown several health benefits such as anti-allergic, antifungal, anti-inflammatory, antimicrobial, and antitumor effects [32]. All these properties would make it possible to turn the products to which they are added into functional products, and even associate them with the concept of a clean label [34,35]. This responds to the current demands of consumers for fresh, natural, and safe products, satisfying the nutritional requirements and improving the sensorial quality of food products in a sustainable way and without the use of synthetic preservatives [30,36,37,38]. Therefore, based on their activities, the recovery and application of BACs from different plant matrices would be an alternative to synthetic additives in the food industry.

## 3. Innovative Green Extraction Technologies

The effectiveness of extracts obtained from plants depends largely on their extraction process, where the initial material, the extraction technique, the solvent, and the processing conditions (temperature and extraction time) used are among the factors that influence their antioxidant activity [23].

The conventional extraction methods are still the most widely used to extract BACs from plant material. However, the aggressive processing conditions used could compromise the quality and biological activity of the compounds obtained, leading us to seek new techniques that are closer to the concept of “green” technologies [30]. In this regard, eco-innovative technologies emerge with the aim of developing techniques characterized by the synthesis of safe products (minimize or eliminate the use of non-toxic solvents), faster extraction rate, more effective energy use, increase in mass and heat transfer, reduction in equipment size and in the number of processing steps, and preserving the natural environment and resources [39]. Accelerated solvent extraction (ASE), enzyme-assisted extraction (EAE), high hydrostatic pressure extraction (HHPE), infrared-assisted extraction (IAE), microwave-assisted extraction (MAE), pulsed electric field extraction (PEF), subcritical fluid extraction (SFE), and ultrasound-assisted extraction (UAE) or the combination of some of them are among the most outstanding techniques [23].

Therefore, the current trend is to replace potentially harmful organic solvents (hexane, benzene, methanol, chloroform, petroleum ether, and acetone) with non-toxic or food-safe ones [40,41]. Green solvents as water, aqueous ethanol solutions, natural deep eutectic solvents, and supercritical fluids are preferred for extraction processes [42]. Table 1 shows some applications of the use of these solvents for the extraction of phenolic compounds from plants.

Water, used since ancient times in decoction, infusion, maceration, and percolation, would be considered the greenest solvent. However, it does not offer good results in the extraction of non-polar or some semi-polar compounds [43]. Its effectiveness could be increased when it is combined with other extraction methods through the use of enzymes, or different temperature and pressure conditions. This last option, known as subcritical water extraction (SWE), could be used for the extraction of essential oils, carotenoids, and phenolic compounds, among others [44].

Another possibility is offered by bio-solvents, mainly represented by ethanol. This solvent, obtained by the fermentation of sugar-rich materials such as sugar beet and cereals, is a good option to replace organic solvents, since for a low price it offers a renewable and biodegradable resource, characterized by being non-toxic and having a high purity and solvent power [45,46]. As an inconvenience, it is important to highlight its difficulty in solubilizing less polar molecules [47].

Carbone dioxide (CO_2_) is the most widely used option for supercritical fluid extraction application, since it is generally recognized as safe (GRAS). Essential oils traditionally extracted by hydrodistillation could be obtained using SFE from seeds, roots, flowers, and leaves, avoiding the use of organic solvents and high temperatures [48]. In addition, the selectivity of this solvent could be adjusted using co-solvents, with ethanol the most widely used due to green requirements [44].

Finally, natural deep eutectic solvents (NADES) are characterized by their chemical and thermal stability, high viscosity, low volatility, and non-inflammability. In addition, they are environmentally friendly and readily biodegradable [49]. The use of amino acids, choline chloride, organic acids (such as citric, lactic or malic acids), and sugars (such as fructose, glucose or sucrose) would allow us to obtain safe compounds such as flavonoids, phenolic acids, peptides, and volatile compounds from natural matrices [42]. Moreover, their combination with techniques such as UAE and MAE would favour BAC extraction.

Once extracted, different stabilisation techniques are used to maintain the bioactive properties of BACs, as well as their quality during the storage. In this way, freeze-drying or encapsulation is optimised for the production of powders and emulsions from the extracts to protect vulnerable components [63]. Encapsulation techniques such as spray-drying can provide efficient alternatives to protect them from harmful environments and also contribute to preserve the nutritive value, bioavailability, solubility, and functionality of BACs, masking off-flavours and odours, controlling their release and their handling in foods [64].

## 4. Application of Plant Extracts in Fresh Meat and Meat Products

**Figure 2:**
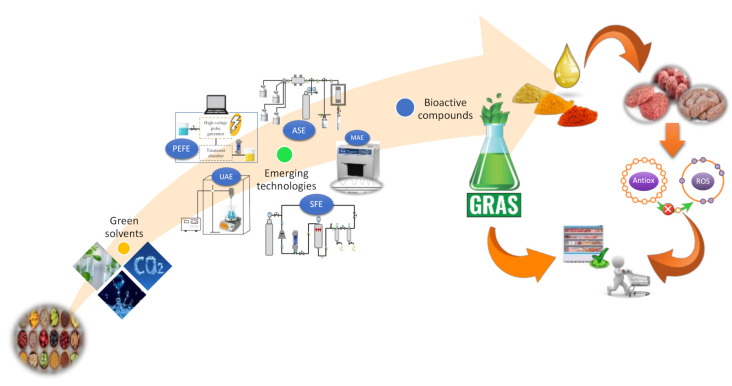
Extraction of BACs by eco-innovative technologies and application as antioxidants in fresh meat and meat products.

The composition of meat and meat products makes them susceptible to oxidation reactions, especially those that involve the degradation of lipids [6]. This makes antioxidants important to preserve the quality of these products. The application of natural antioxidants, especially those containing polyphenols, can be a promising strategy due to their efficacy as scavenging radicals, as well as the additional health benefits they bring to meat products [20]. In this regard, several publications have focused on the use of natural antioxidants (extracted from herbs, spices, fruits, and vegetables using green solvents and/or emerging technologies) in order to decrease lipid and protein oxidation in fresh meat products (Figure 2). These meat products, not subjected to heat treatments, allow us to observe the behaviour of natural antioxidants more clearly during the shelf life of the product, avoiding the effect that temperature could have on antioxidant properties, since many BACs present in the composition of plant extracts are thermolabile.

### 4.1. Plant Extracts Obtained with Green Solvents

As mentioned previously, plants and more specifically cereals, fruits, grains, herbs, seeds, spices, and vegetables are the major sources of natural antioxidants due to their polyphenol contents, which allow us to delay the oxidation processes that occur in meat products during their shelf life [24]. The choice of solvent plays a key role in the extraction of these BACs, as it affects selectivity, extraction method, cost, and safety. In this sense, the Registration, Evaluation, Authorisation and Restriction of Chemicals (REACH) directive has notably incentivized “green” solvents to replace organic ones [47]. Table 2 shows recent applications of natural antioxidants extracted from plants with green solvents in meat and meat products.

Fruits have recently gained great importance due to their health-promoting properties and their antioxidant potential in meat products [65]. This is the case for the fruits of *Myrciaria cauliflora*, which are used as a natural remedy for many diseases as they have anti-inflammatory, antimutagenic, and antimicrobial properties. In addition, their anthocyanins also give them antioxidant properties [66]. These flavonoids are especially present in their peels (dark colour), which are considered a co-product of processing. In this regard, Baldin et al. [66] evaluated the application of aqueous extracts of peels and seeds as natural antioxidants in fresh pork sausages. The results reflected the feasibility of using these extracts as antioxidant and natural pigments, which would avoid the use of colouring agents such as cochineal carmine. During lipid oxidation, TBAR values of sausages treated with 2% and 4% of jabuticaba extract displayed very low values compared to those found in control samples after 15 days of storage (0.01 and 0.02 mg MDA/kg vs. 0.60 mg MDA/kg, respectively). This positive effect was also observed in colour parameters such as a* values, only exceeded by carmine pigments (5.8 and 6.4 vs. 2.8 and 8.0 for 2% and 4% of jabuticaba extract vs. control and samples treated with carmine, respectively). However, the purplish hue of the extract resulted in low scores by consumers. This was not the case with the rest of the attributes, which showed similar values to those observed in the control sample, especially when the lowest dose was used.

Another fruit with high contents of BACs is pomegranate. Its peel, which represents 40–50% of the total fruit weight, is usually discarded during its processing. However, its phenolic acid, flavonoid, and tannin contents makes it an excellent source of valuable compounds. In this regard, several studies confirmed that pomegranate peel extracts improve the quality of meat products during their shelf life [21]. This is the case for beef meatballs, where its potential application as a natural antioxidant displayed even better results than BHT [72]. In addition, at present and in the context of a circular economy, the aforementioned co-products become very important because they represent a renewable and underexploited source with a large amount of phytochemicals with promising bioactive properties [74].

Vegetables are also a good source of phenolic compounds. This is the case of kimchi, a Korean traditional fermented food of various vegetables. Lee et al. [70] evaluated the effect of various kimchi ethanolic extracts: baechu (Chinese cabbage with red pepper—T-BKE), got (mustard leaf, *Brassica juncea*—T-GKE), puchu (scallion, *Allium fistulosum*—T- PKE), and white kimchi (Chinese cabbage without red pepper—T-WKE) on lipid oxidation and the colour stability of raw ground pork meat during refrigerated storage. The incorporation of these extracts at a dose of 1 g/kg resulted in higher redness values than those obtained in control samples at the end of the storage period, especially for extracts that contained red pepper (14.90, 13.83, 13.27 and 12.24 vs. 12.19 for T-BKE, T-GKE, T-PKE, T-WKE, and the control, respectively). These results were confirmed with those obtained in the total colour difference. In all cases, the values obtained were higher than the values considered noticeable (ΔE* > 2, [75]), and treated samples displayed the lowest values; T-GKE and T-WKE presented values closer to this threshold. In addition, kimchi treatments were very useful in the inhibition of MetMb formation. The values found in kimchi treatments were closer to the discolouration limits (40%) capable of being detected by consumers. In this case, T-BKE extracts again showed the lowest changes in MetMb (40.79 vs. 66.99 for T-BKE and control, respectively). Regarding lipid oxidation, the values obtained were below the limit level of deterioration (0.6 mg MDA/kg) for the rancid flavour in meat products [76]. Among the extracts used, GKE and PKE were the most effective. Therefore, this study suggests that the tested kimchi extracts, especially GKE, have potential as a natural preservative to reduce colour degradation, lipid oxidation, and bacterial counts of raw ground pork meat.

Another plant rich in polyphenols, especially flavonoids, is epazote (*Chenopodium ambrosioides* L.). This plant, native to Mexico, has been traditionally used in folk medicine through the infusions of several parts of the plant. Villalobos-Delgado et al. [68] transferred its use to the food industry through the incorporation of the ethanolic extracts of its leaves, flowers, and stems in raw ground pork. ρ-coumaric acid and quercetin were the main phenolic compounds identified in the extract, although in a smaller content, kaempferol 3-O-rutinoside, kaempferol O-rhamnosyl-pentoside, and quercetin dirhamnoside were also quantified. These compounds could explain the protection against rancidity observed in the samples treated with epazote extract, which displayed TBAR values below 0.40 mg MDA/kg. A lower discolouration of the product was also observed, which showed higher a* values (3.35 vs. 1.85, for epazote and control samples, respectively). The protective role of the epazote extract was also observed in sensorial analysis, since it prevented the deterioration of the organoleptic quality of the product, especially observed in the odour scores (3.61 vs. 2.57, for epazote and control samples, respectively). Similar results were observed when the extracts were applied to ground beef [69].

Regarding spices, black pepper (*Piper nigrum* L.) is known as one of the most popular flavouring spices in the world. Its dried berries are widely used in meat products as additives due to the pungency of its extracts and the aroma of its EOs [23]. Zhang et al. [67] reported that black pepper EO applied at concentrations of 0.5% maintained the lipid stability of fresh pork loins during nine days of storage, reducing the TBAR values and delaying MetMb formation. In addition, treated samples had higher activity against Gram-negative bacteria. Its antioxidant and antimicrobial activities are due to the presence of notable contents of caryophyllene, limonene, α-terpinene, and α-pinene [77]. The positive effects of the EO were also observed in TVB-N, considered a marker of meat quality and freshness. The values obtained were lower in treated samples and were below the limit values for pork (26 mg of nitrogen/100 g of muscle) [78,79].

The products of beekeeping (pollen and propolis), an activity strongly linked to sustainability, are known for their beneficial effects on health such as anti-inflammatory, antihypertensive, antidiabetic, and antimicrobial effects. In addition, their contents in phenolic compounds give them an important antioxidant activity [80]. There are several studies in meat products that have shown their positive effect in the reduction of lipid oxidation, thus preventing a decrease in the sensory quality and in the nutritional value of these products. Bee pollen is an agglomerate of flower pollen from various botanical sources, which are collected by the bees and mixed with nectar and honeybee salivary substances, carried out by worker bees, and collected at the hive’s entrance. Bee pollen consists of substances that are nutritionally essential, such as amino acids, vitamins, macronutrients, and micronutrients, as well as polyphenols [18,81]. Anjos et al. [18] studied the effect of lyophilized ethanolic (80%) bee pollen extracts on the shelf life of black pudding. They observed that the degree of lipid oxidation decreased from 3.02 mg MDA/kg at the beginning of storage to 1.27 mg MDA/kg after 30 days of storage, similar to the values found in the black pudding prepared with commercial antioxidants (1.27 mg MDA/kg). This is probably due to the presence of quercetin derivatives in the composition of the extracts. In addition, sensorial analysis revealed that their incorporation in the product did not affect the preference of the consumers. Therefore, bee pollen extract could be used as a natural antioxidant in meat products, always taking into account the possible allergy risks that it could have for some consumers. These results were corroborated by those found by de Florio Almeida et al. [9], who reported the strong antioxidative effects of a lyophilized bee pollen extract on lipid oxidation of pork sausages. Kaempferol, quercetin, *trans*-cinnamic acid, and ρ-coumaric acid are responsible for its activity, reflected in the values obtained from TBARS at the end of storage that were similar to those found in the samples treated with sodium erythorbate (4.08 and 3.88 mg MDA/kg vs. 4.71 mg MDA/kg, for bee pollen extract, erythorbate, and control samples, respectively). Despite these positive effects, the values found were higher than those considered acceptable for this oxidation marker (2.5 mg MDA/kg) [82].

Propolis, a substance resulting from moistening the material that bees collect from resinous and pollen material with saliva and enzymatic secretions and with wax, also displayed antioxidant effects on the oxidative stability of fresh patties [73]. The presence of the flavonoids, pinocembrin, naringenin, and galangin in the propolis ethanol extract is responsible for its antioxidant properties, resulting in the reduction of lipids, protein oxidation, and colour loss during nine days of storage. In treated samples, the values of the parameters evaluated were below the threshold values of acceptability. In this regard, the values of TBARs and carbonyls were less than 0.5 mg MDA/kg and 1.5 nM carbonyl/mg, respectively. The same occurred with MetMb contents, which at the end of storage were below 40%, resulting in higher redness values (15.0 vs. 9.2, 8.1 and 9.5, for beef patties with propolis extract, control, and samples with BHT (0.02%) and ascorbic acid (0.015%), respectively; 15.7 vs. 11.6, 11.1 and 10.2, for pork patties with propolis extract, control, and samples with BHT (0.02%) and ascorbic acid (0.015%), respectively).

These results confirmed the effectiveness of beekeeping products for oxidative stability of meat products. In a similar way, lotus (*Nelumbo nucifera*), used since ancient times in Chinese folk medicine due to the medicinal properties of its roots (anti-anxiety, antifungal, and anti-inflammatory activities) and its leaves (relieve fever and improve body energy), contains abundant levels of polyphenolic compounds that confirm its antioxidant activity. In this regard, the use of lotus (*Nelumbo nucifera*) root and leaf ethanol extracts (0.1, 0.5, and 1.0%) retarded the lipid oxidation reactions in pork patties, being the extract of lotus leaves the extract that presented the highest antioxidant activity probably due to flavonoids, phenolic acids, and tocopherols, which are among the compounds present in its composition [71]. These results are in agreement with those observed by Choe et al. [83] for cooked ground pork containing lotus leaf powder, which displayed lower POVs, CD, and TBARS compared to those found in control samples. Nevertheless, the greenish colours of applied extracts resulted in lower values of a * (3.1 and 3.3 vs. 3.8 for 1.0% leaves and root extracts vs. BHT and control samples, respectively), an effect also observed in sensory assessment.

Regarding dose effect, concentrations higher than 0.5% resulted in TBARS values below 1 mg MDA/kg shown by BHT. In the case of leaf extracts, the values were even below the limit for the sensory perception of rancidity in meat products (0.6 mg MDA/kg). The synergistic effect of both extracts (0.5% lotus root extract and 0.5% lotus leaf extract) displayed positive effects both on primary and secondary lipid oxidation products, probably due to the difference in antioxidizing mechanisms, since root extracts showed a significantly higher chelating activity and leaf extracts displayed higher reducing power and DPPH radical scavenging activity. However, the synergistic effect did not result in better results than those obtained with the highest concentration of the leaf extract. These outcomes were also observed in the overall acceptance scores obtained (7.1 for 0.5% root-leaf extracts and BHT vs. 6.9, 6.8, and 6.8 for 1.0% leaf extracts, the control and 1.0% root extracts).

### 4.2. Plant Extracts Obtained by Emerging Technologies

Fruits and vegetables are considered an important source of natural antioxidants, which is related to their high contents of phenolic compounds [65]. The extraction of these BACs largely depends on the effectiveness and efficiency of the selected extraction methods, with emerging technologies being those that allow us to overcome disadvantages of conventional extraction ones (Table 3). This is the case of red dragon fruit (*Hylocereus monacanthus*) whose antioxidant capacity is mainly due to its betalain content, followed by its biosynthetic precursors and phenolics such as gallic acid and acetylcoumarin [84]. Bellucci et al. [37] evaluated the possibility of obtaining an extract from the pulp of this fruit using PEF technology. The application of PEF resulted in an extract with a good antioxidant capacity, even higher than the results obtained by other authors with conventional procedures (825.40 μmol Fe^+2^/100 g and 229 mg Trolox/100 g, for FRAP and DPPH values, respectively). This activity was also reflected when this extract (250, 500, and 1000 mg/kg) was incorporated in a meat product. Although the results of lipid and protein oxidation did not improve those obtained by erythorbate, its application allowed extension of the shelf life of pork patties during the storage time. Regarding colour parameters, the intense pink colour of the extract enhanced the colour stability, displaying higher a* values as the concentration increased (9.33, 7.92 and 7.69 vs. 6.77 and 6.60, for high, medium, and lower doses vs. control and samples treated with erythorbate, respectively). This result was also observed in the total colour variation at the end of storage, although noticeable (ΔE* higher than 2) values were lower than those observed in control and erythorbate treatments (4.20, 4.29 and 4.81 vs. 5.36 and 6.11, for high, medium, and lower doses vs. control and samples treated with erythorbate, respectively). Regarding sensorial analysis, pitaya extract did not have an effect on odour, taste, texture, and overall acceptance, but the highest scores obtained for colour attributes resulted in the best results for these samples in the preference test, since colour is the main attribute evaluated by consumers when choosing a meat product [85].

The seeds of the fruits produced by guarana (*Paullinia cupana*) can also be included in this source of natural antioxidants, since they contain tyrosols, proanthocyanidins, and (epi)catechin [96]. Guarana seed extracts have been studied as natural antioxidants to inhibit lipid oxidation of burgers. The extraction of the plant material consisted of its dispersion in a hydroethanolic solvent (40:60, water: ethanol) combined with a UAE treatment for 45 min. The application of this extract in a powder form at different concentrations (250, 500, and 1000 mg/kg) in pork patties resulted in promising results against lipid and protein oxidation during refrigerated storage [87]. The effect on colour showed that the highest dose allowed the product to remain colour stable (a* values) for 15 days, as well as prevent MetMb formation (55.8% vs. 60.6% and 57.2%, for GSE, control, and BHT, respectively). A different trend was observed in TBAR values and carbonyl contents, since lower and medium doses were sufficient to delay oxidation, showing even lower values than those observed in BHT samples. These outcomes are corroborated with those obtained for raw lamb burgers [14]. In this case, guarana seed and pitanga (*Eugenia uniflora* L.) leaf extracts added at concentrations of 250 mg/kg delayed the discolouration of the burgers and retarded lipid and protein oxidation during storage. It is worth highlighting the results showed by pitanga extracts, which presented the lowest TBAR and carbonyl values, without impairing the sensorial characteristics. In addition, the changes in the secondary products of lipid oxidation were also evaluated through volatile compounds. The contents of hexanal, the best indicator of lipid oxidation, were lower in samples treated with these natural extracts (4.79 and 9.76 vs. 18.92 and 112.75 AU × 10^4^/g, for pitanga, guarana, BHT, and control samples, respectively). Therefore, these extracts represent a promising alternative to extend the shelf life of meat products, replacing synthetic antioxidants by natural ones.

The leaves of the genus *Rosmarinus*, commonly used in traditional medicine, are considered a natural source of antioxidants, associated with the presence of BACs, mainly carnosol and rosmarinic acid [97]. The products obtained from *Rosmarinus officinalis* L. are regulated by the FDA. In this regard, several authors reported the promising use of rosemary (*Rosmarinus officinalis* L.) extract to extend the shelf life of meat products. Wang et al. [92] evaluated the physicochemical stability of omega-3 fatty-acid-fortified surimi-like meat products with ethanolic extracts of rosemary. The authors observed that their addition during the chopping procedure would prolong the shelf life of these type of products in the industry, decreasing lipid oxidation markers such as POV, CDs, and TBARs. Positive effects were also obtained with the use of ethanolic extracts of mesquite leaves (*Prosopis velutina*). Ramírez-Rojo et al. [90] studied the effect of the extract (0.05 and 0.1%), obtained by UAE (42 kHz/25 °C/30 min) on the preservation of pork patties stored for 10 days at 4 °C. The authors reported that this ethanolic extract could be used to extend the shelf life of meat products, showing a better stability of colour, MetMb, and lipid oxidation during storage. The polyphenol content of this extract (hydroxycinnamic acid, anthocyanin, tannin, and flavonoids) could be the responsible for the quality improvement at the end of the storage period, resulting in a product with a better sensory acceptability.

The leaves of *Artemisia afra* and *Bidens pilosa* have also been used in traditional medicine for the treatment of different diseases [98,99], but they have also been proposed as alternatives to synthetic additives in the meat industry with the aim of developing healthy and safe meat products. In this way, Falowo et al. [86] studied their application in ground pork, a product very susceptible to oxidation, since during grinding there is a breakdown of the muscle membranes, and the reactions between pro-oxidant molecules and unsaturated compounds enhance the formation of free radicals and the propagation of oxidative reactions [100]. The application of the EO obtained by solvent-free MAE resulted in lower rates of lipid oxidation, especially in the samples with *A. afra* (1.12 and 1.42 mg MDA/kg vs. 1.46 mg MDA/kg for *A. afra* and *B. pilosa* vs. the control sample, respectively). These results are probably due to the higher content and potent BACs (thujone, eucalyptol, and camphor) identified in *A. afra* EO than those found in *B. Pilosa* (caryophyllene, humulene, and γ-elemene).

The aerial parts of the plant *Satureja montana* L. (winter savory), the most commonly used of the *Satureja* genus, have been used since ancient times for their antimicrobial and antioxidant effects [33]. Šojić et al. [94] demonstrated the successful application of the supercritical extract obtained from the aerial parts of this plant in fresh pork sausages. The authors suggested that the co-extracted lipids present in the extract resulted in an increase in the antioxidant activity, probably linked to the carvacrol and ρ-cymene contents. In addition, these extracts obtained using an eco-innovative technology contributed to the sensory quality improvement, which is an advantage over conventional technologies such as hydrodistillation, where extracts provided strong flavours and odours to the product.

Roots are also a natural source of antioxidants. This is the case of the rhizomes of ginger (*Zingiber officinale*) or turmeric (*Curcuma longa* L.), which can be used as potential alternatives to synthetic antioxidants in meat products. de Carvalho et al. [38] evaluated the application of different doses of supercritical turmeric extract in fresh lamb sausages. The results found were promising since its use increased the antioxidant capacity of lamb sausages (418.4, 292.9 and 140.3 μg Trolox/g vs. 91.6 and 87.1 μg Trolox/g, for turmeric extract added at 250, 500, and 750 mg/kg vs. control and erythorbate samples, respectively), which resulted in a reduction in lipid oxidation (0.44, 0.49, and 0.68 mg MDA/kg vs. 2.76 and 1.57 mg MDA/kg turmeric extract added at 250, 500, and 750 mg/kg vs. control and erythorbate samples, respectively) and low release of lipid-derived volatile compounds, especially in the case of hexanal considered as a marker of lipid oxidation (5.44, 2.68 and 11.02 AU × 10^4^/g of sample vs. 72.62 and 85.43 AU × 10^4^/g of sample), even at lower doses than those used for synthetic antioxidant (sodium erythorbate). Moreover, physicochemical parameters (cooking loss and texture profile) and sensorial attributes (colour, discolouration, and odour) were not greatly influenced by turmeric addition.

Agro-industrial by-products can also be used to obtain potential natural antioxidant extracts. In this regard, the ethanolic extract obtained from the stems, leaves, and rejected pink peppers of the plant *Schinus terenbithifolius* Raddi was applied to extend the shelf life of chicken restructured product [101]. This extract, characterized by notable catechin, ρ-coumaric acid, myricetin, and epicatechin contents, resulted in the delay of lipid oxidation both when applied directly to the meat batter and through its incorporation into chitosan films. This could probably be related to its phenolic content. Saldaña et al. [19] corroborated the effect of pink pepper residue extract in chicken burger, since the scores obtained were similar to those obtained by comparison with commercial samples.

Another industrial by-product that could be used as a natural preservative for food industry is sage (*Salvia officinalis* L.) herbal dust. This medicinal plant, belonging to the *Lamiaceae* family, is recognized for its biological activities since it has notable contents of BACs [102], especially rosmarinic acid, carnosic acid, and carnosol [23]. In addition, its EO contains terpene compounds such as α-and β-thujone, camphor, and eucalyptol [103]. For these reasons, it can be considered that its by-product generated in filter tea factories could also have a high content of BACs in its composition and therefore, high antioxidant activity. In addition, regarding the safety of its use, there are no studies related to its toxicity, and it is regulated by the FDA [104]. Śojić et al. [93] studied the effect of sage by-product extract obtained by SFE on lipid oxidation of fresh pork sausages. The authors found that this extract had a good antioxidant potential, showing the highest inhibitory potential at a concentration of 0.1 μL/g and providing better sensory properties of fresh pork sausages, which suggests the advantage of using this alternative extraction technique.

By-products of powdered herbal material resulting from fractionation of wild thyme (*Thymus serpyllum* L.) in the herbal infusion industry could also be used as antioxidants to improve the quality of meat products during their shelf life. Their activity is associated with the presence of terpinoids (carvacrol, thymol, and α-terpineol), flavonoids, and tannins, among others, in their composition [105]. In this regard, Šojić et al. [95] demonstrated the positive effect of the application of supercritical extracts (0.075 and 0.150 µL/g) of wild thyme by-products in ground pork patties. The samples containing wild thyme extract displayed a significant reduction in lipid oxidation, with values below the flavour deterioration threshold (0.6 mg MDA/kg) in meat products [76]. The presence of monoterpene polyphenols also prevented discolouration in samples with wild thyme extracts, showing the highest a* values at the end of storage compared to those observed in the control (8.91–9.54 vs. 7.64, respectively). The hydroxyl groups of thymol and carvacrol, the major terpenoids identified in the extracts, could be responsible for the reduction of protein oxidation observed in the samples with natural antioxidants, since they are capable of donating an electron in order to neutralize free radical reactions [87].

## 5. Conclusions and Future Trends

The results found in the bibliography confirm that the use of plant extracts can extend the shelf life of fresh meat products, delaying the deterioration of the products, and thus maintaining their organoleptic characteristics. In addition, the health-related characteristics associated with these BACs results in a meat product that contains them that is a functional product, which would help to alleviate human dietary deficiencies. This strategy allows us to meet the current consumer demands, since it is known that health and nutrition go hand in hand. Therefore, their technological properties and their health promotion effects (prevention of diseases) together with their natural origins and the absence of undesirable secondary effects makes them potential substitutes for synthetic antioxidants. Moreover, these products could also be classified as clean due to the replacement of synthetic additives by natural ones, thus avoiding the presence of negatively perceived ingredients (additives, allergenic ingredients, or those perceived as unfamiliar and chemical-sounding).

On the other hand, the need to obtain more ecological, sustainable, and viable extraction processes has led food industries and scientists to develop alternative processes according to the “green” extraction concept. These processing schemes allow us to extract and process high-added-value ingredients with proven health biological activities and that are safer for consumers. In addition, these technologies allow the valorisation of the co-products obtained from agri-food resources, contributing to the sustainability of the food chain and reducing the environmental impact of food production due to the better use of local materials. In addition, their incorporation into food would contribute to improve both technological and functional characteristics, thus diversifying the offer of so-called “wellness foods”.

Along with these strategies, the recommendations of international organizations to reduce the consumption of some nutrients (nitrates and nitrites, saturated fats, *trans*-fats, and salt) are becoming increasingly important, forcing industries to reformulate meat products to obtain healthier meat products. Therefore, the combination of these strategies is important to align the production of healthy meat products with sustainable actions.

Many of these technologies mentioned have already been scaled up to an industrial level. However, although the use of natural extracts is becoming more and more common, their industrial use does not seem to be imminent. Even though many of these compounds have passed strict controls and are Generally Recognized as Safe (GRAS), there is still no regulations that legislate the inclusion of these compounds in meat products. In addition, prior to incorporation in meat products, it is also necessary to establish the optimal dose and evaluate the toxicity of these extracts, as well as their bioaccessibility and bioavailability in the human body.

## Figures and Tables

**Table 1:** Extraction of phenolic compounds from plant sources using green solvents and technologies.

Extraction Technique	Plant Source	BACs	Extraction Conditions	Ref.
SFE	Lavender (*Lavandula angustifolia* Mill.)	Oxygenated monoterpenes, coumarin, herniarin	40–60 °C, 100–300 bar, 1–3 kg/h CO_2_, 90 min	[50]
	Marjoram (*Origanum majorana*) Oregano (*Origanum vulgare*)	*cis*-Sabinene hydrate, linalyl acetate, terpinen-4-ol, α-terpineol *cis*-Sabinene hydrate, thymol, carvacrol, terpinen-4-ol, geraniol,	40 °C and 200 atm 180 min	[51]
	Savory	Carvacrol	40–60 °C, 100–350 bar, 4.5 h, 0.194 kg/h CO_2_	[52]
SWE	Arctostaphylos *uva-ursi* herbal dust	Phenols and flavonoids	120–220 °C, 10–30 min, 30 bar, HCl: 0–1.5%	[53]
	Ginger (*Zingiber officinale*)	Gingerol	130–140 °C, 10–40 min, 2 bar	[54]
	Tumeric rhizomes (*Curcuma longa* L.)	Curcumin	120–160 °C, 6–22 min, 10 bar	[55]
	Wild garlic (*Allium ursinum* L.)	Phenols and flavonoids	120–200 °C, 10–30 min, HCl: 0–1.5%	[56]
	Winter savory (*Satureja montana* L.)	Phenols and flavonoids	79.15–220.5 °C, 5.9–34.1 min, 30 bar	[57]
NADES	Blackcurrant (*Ribes nigrum* L.)	Anthocyanins	ChCl:La MAE (15 min, 45 °C)	[58]
	Elderberry plant (*Sambucus nigra*)	*Phenolic acids*: neochlorogenic acid, chlorogenic acid, di-caffeoylquinic acid, *p*-coumaroylquinic acid derivative *Flavonols*: quercetin 3-*O*-rutinoside (rutin), quercetin 3-*O*-glucoside (isoquercitrin), isorhamnetin-3-*O*-rutinoside, quercetin	La:Gln UAE (50 Hz and a power of 550 W, 5–40 min, 40–80 °C)	[59]
	*Moringa oleifera* L.	*Phenolic acids*: gallic acid, *p*-hydroxybenzoic acid, rosmarinic acid, *Flavonoids*: (+)-catechin, vicenin-2, orientin, rutin, hyperoside, kaempferol-3-*O*-rutinoside, isorhamnetin 3-*O*-glucoside, quercetin, apigenin, kaempferol, (−)-epigallocatechin	L-proline:Gly UAE (15 min, 40 °C, 144 W)	[60]
	Onion, olive, tomato and pear industrial by- products	Gallic acid, 3-Hydroxytyrosol, Tyrosol, Catechin, Caffeic acid, Rutin, Coumaric acid, Trans-Ferulic acid, Ooleuropein, Cinnamic acid, Quercetin, Luteolin, Naringenin, Apigenin	LaGlc UAE (0–60 min, 40 °C, 200 W)	[61]
	Rosemary (*Rosmarinus officinalis* L.)	*Phenolic acids*: rosmarinic acid and ferulic acid *Flavonoids*: 7-methylrosmanol, rutin, naringin	ChCl:ProOH UAE (120 min, 40 °C)	[62]

ChCl: choline chloride; Glc: glucose; Gln, glycine; Gly, glycerol; La: lactic acid; Pro: L-proline; ProOH: 1,2-propanediol.

**Table 2:** Application of natural antioxidants extracted with green solvents from plants in meat and meat products.

Plant Source	Extraction Method	Phenolic Content and Antioxidant Activity	Extract Dose	Meat Model System	Storage Conditions	Main Effects	Ref.
Black pepper (*Piper nigrum*)	Anhydrous ethanol	n.d.	0.1 and 0.5% (*v/v*) in 20%	Fresh pork	9 days at 4 °C	Dose-dependent effectiveness. Delay in MetMb formation, resulting in higher L* and a* values. Halving of TBAR values. Lower TVB-N (below limit values for pork).	[67]
Bee pollen (*Cistus ladanifer* pellets)	80% EtOH-water (*v/v*)	TPC: 35.05 mg GAE/g; TFC: 6.99 mg QE/g; DPPH (IC_50_): 2.62 mg/mL; RP: 6.51 mg GAE/mL	−	Black pudding	37 days at 4 °C	Healthy food product. Improves quality and consumer acceptance	[18]
		TPC: 19.69 mg GAE/g; TFC: 6.81 mg QE/g; DPPH (IC_50_): 0.97 mg/mL; DPPH: 54.42 of radical inhibition; ABTS: 120.10 µmol TEAC/g; FRAP: mmol Fe^2+^/g; β-carotene/linoleic acid: 91.93%	0.2%	Pork sausages	30 days at 4 °C	Higher protection against Lox (13.4% of inhibition).	[9]
Epazote (*Chenopodium ambrosioides* L.)	Water Ethanol	TPC: 126.3 mg GAE/100 g; TFC: 147.26 mg QE/100 g; DPPH (IC_50_): 0.97 mg/mL; DPPH: 16.65% of radical inhibition	50 mL/kg	Raw ground pork	9 days at 4 °C	Protective role against deteriorating processes (Lox and myoglobin stability) during storage. Good acceptability scores.	[68]
			0.5 g/50 mL	Raw ground beef		Inhibited Lox and received the highest score in sensorial attributes evaluated.	[69]
Jabuticaba (*Myrciaria cauliflora*) peels and seeds	Water ME	TPC: 15.63 mg GAE/g; Anthocyanin content: 7.21 mg CE/g; FRAP: 20.51 µmol TE/g; DPPH: 52.90 mmol TE/g.	2 and 4%	Fresh pork sausages	15 days at 1 °C	Lower TBAR values. Higher dose negatively influenced sensory attributes.	[66]
Kimchi	75% EtOH	n.d.	1 g/kg	Ground pork meat	14 days at 4 °C	Positive effect on colour, displaying lower deterioration (ΔE* and MetMb). Lower TBAR values (<0.4 mg MDA/kg).	[70]
Lotus (*Nelumbo nucifera*) root and leaf	Ethanol	DPPH (IC_50_): 0.52 and 0.17 g/L for lotus root and leaf, respectively; Chelating activity (IC_50_): 0.32 and 0.84 g/L	1% (*v/v*)	Pork patties	10 days at 4 °C	Leaf extract was preferred to root extract. Reduction of primary and secondary oxidation. Greenish colours and indigenous taste of extracts gave rise to adverse effects on colour and flavour scores, while combined extracts resulted in highest overall acceptance scores.	[71]
Pomegranate (*Punica granatum* L.) peel extract	Concentrated lyophilised water extract	TPC: 165.4 mg GAE/g; FRSA: 5720 mM TE/g	0.5 and 1%	Beef meatballs	8 days at 4 °C	The high phenolic content of pomegranate peel extract resulted in lower TBAR values, peroxide and protein carbonyl formation, and loss of sulfhydryl groups. Improvement of sensory scores	[72]
Propolis (*Prosopis velutina* and *Mimosa distachya*)	Ethanol	At 100 µg/mL TPC: 198.5 mg GAE/g; RPA (100 µg/mL): 0.20 abs; FRSA: 33.0%	2% (*w/w*)	Beef and pork patties	9 days at 2 °C	The richness in phenolic compounds resulted in higher colour stability and inhibitions of Lox and Pox.	[73]

a*: Redness; abs: absorbance measured at 700 nm; ABTS: 2-2′-Azino-di-[3-ethylbenzthiazoline sulfonate] Radical Scavenging Activity; ΔE*: Total colour difference; DPPH: 2,2-diphenyl-1-picrylhydrazyl Radical Scavenging Activity; FRAP: Ferric Reducing Antioxidant Power Assay; CE: cianidin-3-glucoside equivalent; FRSA: Free-radical scavenging activity; GAE: gallic acid equivalent; IC_50_: Half maximal inhibitory concentration; L*: Lightness; Lox: Lipid oxidation; MDA: malonaldehyde; ME: Microencapsulated; MetMb: Metmyoglobin; n.d.: not determined; Pox: Protein oxidation; QE: quercetin equivalent; RPA: Reducing power assay; TBARS: Thiobarbituric acid reactive substances; TE: Trolox equivalent; TEAC: Trolox Equivalent Antioxidant Capacity; TFC: Total flavonoid content; TPC: Total phenolic content; TVB-N: Total volatile basic nitrogen.

**Table 3:** Application of natural antioxidants extracted with emerging technologies from plants in meat and meat products.

Plant Source	Extraction Method	Phenolic Content and Antioxidant Activity	Extract Dose	Meat System	Storage Conditions	Main Effects	Ref.
*Artemisia afra* and *Bidens pilosa*	SFME	DPPH (400 µL): 68.26% and 13.91% radical inhibition of *Artemisia afra* and *Bidens pilosa*, respectively	0.2 mL/100 g	Pork patties	7 days at 4 °C	Treated samples displayed lower TBARs, even better than BHT. *A. afra* EO exhibited higher activity than *B. Pilosa*.	[86]
Guarana seed (*Paullinia cupana*)	Hydroethanolic Solvent—UAE	TPC: 258 mg GAE/g; DPPH: 0.3 g/L; TEAC: 2072 μmol TE/g	250 mg/kg	Lamb burgers	18 days at 2 °C	Potential substitutes for BHT that even improved the results of Lox and Pox. Hexanal contents corroborated their positive effect on lipid oxidation, especially in pitanga leaf extracts. Delay in MetMb contents with redder intensity of treated samples.	[14,87,88]
Pitanga leaves (*Eugenia uniflora* L.)		TPC: 229.38 mg GAE/g; DPPH: 242 μg/mL; ABTS: 570.97 mg TE/g					
Lemongrass (*Cymbopogon citratus*)	Cereal alcohol (70%)—UAE	TPC: 133.84 mg GAE/g; TFC: 13.42 mg QE/g; IC_50_: 0.45 mg/mL	0.5 and 1.0%	Fresh chicken sausage	42 days at 4 °C	Efficient action in combating Lox. Good acceptability by the consumer.	[15,89]
Mesquite leaves (*Prosopis velutina*)	UAE Ethanol	TPC: 278.50 mg GAE/g; TFC: 226.8 mg RE/g; DPPH (100 µg/mL): 85.3% radical inhibition	0.05 and 0.1 % (*w/w*)	Pork patties	10 days at 4 °C	Decrease in Lox. 40% inhibition of CDs and 90% of TBAR values. No significant differences in sensory attributes.	[90]
Oak wood (*Quercus alba*) chips	ASE—Subcritical water	TPC: 2180.8 mg GAE/L; ABTS: 32.00 mM TE/L; DPPH: 31.20 mM TE/L	0.05, 0.5 and 1.0%	Pork patties	12 days at 4 °C	Lower Lox (TBARs and volatile compounds). Improved sensory quality (new attributes: oak and sweet spices).	[91]
Pink pepper residue (*Schinus terenbithifolius* Raddi)	UAE Ethanol	TPC: 45.01 mg GAE/g; ABTS: 931.00 µmol TE/g; DPPH: 535.74 µmol TE/g; ORAC: 158.24 µmol TE/g; IC_50_: 1.24 mg/mL	90 mg GAE/kg meat	Chicken burger	−	Pronounced effect on the sensory characteristics	[19]
Red pitaya (*Hylocereus monacanthus*)	PEF	TPC: 268.13 mg GAE/100 g; FRAP: 825.40 Fe^+2^/100 g; DPPH: 229 mg TE/100 g	250, 500 or 1000 mg/kg	Pork patties	18 days at 2 °C	Enhancement of colour stability (intense pink colour), lipid and protein protection from oxidation, and improvement of colour acceptance and preference	[37]
Rosemary (*Rosmarinus officinalis* L.) leaves	80% EtOH (*v/v*) UAE	TPC: 24.46 mg/g; TFC: 38.36 mg/g; TDTC: 88.76 mg/g	200 mg/kg	Chicken surimi	14 days at 4 °C	Decrease in oxidation markers (POV, CDs, and TBAR values).	[92]
Sage (*Salvia officinalis* L.) by-product	SFE	DPPH (IC_50_): 0.0242 mg/mL	0.05, 0.075 and 0.1 μL/g	Fresh pork sausages	8 days at 3 °C	Synergistic effects of terpenoids and other lipids extracted by SFE, responsible for lower TBAR values. Positive effect on sensory properties.	[93]
Turmeric (*Curcuma longa* L.)		TPC: 5018.42 mg GAE/100 g; ABTS: 1490.53 mg AAE/100 g; DPPH: 42.92 mg TE/g; FRAP: 980.27 µmol Fe^+2^/100 g	250, 500 or 750 mg/kg	Fresh lamb sausage	18 days at 2 °C	Improved the antioxidant capacity of sausages, slowing Lox (lower TBARS and volatile compounds)	[38]
Winter savory (*Satureja montana* L.)		DPPH: 26.17–27.87 µg/mL	0.075 and 0.150 µL/g	Fresh pork sausages	10 days at 3 °C	Improved oxidative and microbial stability. Overall acceptance.	[94]
Wild thyme (*Thymus serpyllum* L.) by-product		ABTS: 576.7–665.6 µM TE/g; DPPH: 37.5–58.3 µM TE/g	0.075 and 0.150 µL/g	Ground pork patties	3 days at 4 °C	Significant reduction in Lox and Pox.	[95]

AAE: ascorbic acid equivalent; ABTS: 2-2′-Azino-di-[3-ethylbenzthiazoline sulfonate] Radical Scavenging Activity; ASE: Pressurised liquid extraction; CDs: Conjugated dienes; DPPH: 2,2-diphenyl-1-picrylhydrazyl Radical Scavenging Activity; FRAP: Ferric Reducing Antioxidant Power Assay; FRSA: Free-radical scavenging activity; GAE: gallic acid equivalent; IC_50_: Half-maximal inhibitory concentration; MetMb: Metmyoglobin; Lox: Lipid oxidation; ORAC: Oxygen Radical Absorbance Capacity Assay; Pox: Protein oxidation; POV: Peroxide values; QE: quercetin equivalent; RE: Rutin equivalents; RPA: Reducing power assay; SFME: Solvent-free microwave extraction TBARS: Thiobarbituric acid reactive substances; TDTC: Total diterpene compounds; TE: Trolox equivalent; TEAC: Trolox Equivalent Antioxidant Capacity; TFC: Total flavonoid content; TPC: Total phenolic content.
